# Skeletal Recovery After Weaning Does Not Require PTHrP

**DOI:** 10.1002/jbmr.339

**Published:** 2011-02-01

**Authors:** Beth J Kirby, Laleh Ardeshirpour, Janine P Woodrow, John J Wysolmerski, Natalie A Sims, Andrew C Karaplis, Christopher S Kovacs

**Affiliations:** 1 Faculty of Medicine, Memorial University of NewfoundlandSt. John's, Newfoundland, Canada; 2 Faculty of Medicine, Yale University School of MedicineNew Haven, CT, USA; 3 St Vincent's Institute for Medical Research and Department of Medicine, St. Vincent's Hospital Melbourne, The University of MelbourneFitzroy, Victoria, Australia; 4 McGill University and Jewish General HospitalMontréal, Quebec, Canada

**Keywords:** Pregnancy, Lactation, PTH/PTHRP, Bone Mineralization, Histomorphometry, Knockout, Animal Models/rodent, Growth and Development

## Abstract

Mice lose 20% to 25% of trabecular bone mineral content (BMC) during lactation and restore it after weaning through unknown mechanisms. We found that tibial *Pthrp* mRNA expression was upregulated fivefold by 7 days after weaning versus end of lactation in wild-type (WT) mice. To determine whether parathyroid hormone–related protein (PTHrP) stimulates bone formation after weaning, we studied a conditional knockout in which PTHrP is deleted from preosteoblasts and osteoblasts by collagen I promoter–driven *Cre* (*Cre*^ColI^). These mice are osteopenic as adults but have normal serum calcium, calcitriol, and parathyroid hormone (PTH). Pairs of *Pthrp*^flox/flox^;*Cre*^ColI^ (null) and WT;*Cre*^ColI^ (WT) females were mated and studied through pregnancy, lactation, and 3 weeks of postweaning recovery. By end of lactation, both genotypes lost lumbar spine BMC: WT declined by 20.6% ± 3.3%, and null decreased by 22.5% ± 3.5% (*p* < .0001 versus baseline; *p* = NS between genotypes). During postweaning recovery, both restored BMC to baseline: WT to –3.6% ± 3.7% and null to 0.3% ± 3.7% (*p* = NS versus baseline or between genotypes). Similar loss and full recovery of BMC were seen at the whole body and hind limb. Histomorphometry confirmed that nulls had lower bone mass at baseline and that this was equal to the value achieved after weaning. Osteocalcin, propeptide of type 1 collagen (P1NP), and deoxypyridinoline increased equally during recovery in WT and null mice; PTH decreased and calcitriol increased equally; serum calcium was unchanged. Urine calcium increased during recovery but remained no different between genotypes. Although osteoblast-derived PTHrP is required to maintain adult bone mass and *Pthrp* mRNA upregulates in bone after weaning, it is not required for recovery of bone mass after lactation. The factors that stimulate postweaning bone formation remain unknown. © 2011 American Society for Bone and Mineral Research.

## Introduction

Substantial amounts of calcium are rapidly supplied to lactating mammary tissue to produce calcium-rich milk. Several lines of evidence have shown that mammals meet this demand by resorbing bone and reducing bone mineral content (BMC) during lactation irrespective of dietary calcium intake.(1–3) The lactational losses are greater from trabecular than cortical bone and from the spine compared with the appendicular skeleton.(1–3) Within 2 to 6 months of exclusive lactation, women typically lose 5% to 10% of BMC, whereas teenaged mothers and women lactating twins may lose even more.(1,4) In contrast to humans, lactating rodents are faced with a proportionately greater calcium demand from litters of 6 to 12 pups and will lose 20% to 25% of BMC during 3 weeks of lactation (and even more with larger litters or dietary calcium restriction).(1,5–7) The skeletal losses reach 55% of trabecular BMC in mice that lack the gene encoding calcitonin and calcitonin gene–related peptide α.(6)

The mechanism by which these lactation-induced losses of skeletal mineral content occur has been the subject of recent investigations by our laboratories.(6–12) The mammary glands produce large amounts of parathyroid hormone–related protein (PTHrP). PTHrP is secreted at high concentrations into milk, but some escapes into the maternal circulation with suckling, where it has an impact on systemic mineral homeostasis. Together with low estradiol, PTHrP acts to stimulate osteoclast-mediated bone resorption,(13) and additional stimulatory factors may be implied.(14) It also has been appreciated more recently that osteocytic osteolysis occurs during lactation and likely contributes to the loss of mineral content.(15)

At the end of lactation, osteoclasts undergo apoptosis, and osteoblasts begin to rapidly build bone and remineralize the skeleton.(1,16) Osteocytic lacunae also show tetracycline labeling, confirming that new bone is being laid down at these sites.(17,18) Typically within 6 months in humans and 2 to 3 weeks in mice, the BMC returns to the prepregnancy baseline value,(1–3) even in mice that have lost 55% of spine BMC.(6) This means that the postweaning skeleton gains 2% to 3% BMC per month in humans and 10% to 20% per week in mice. This rapid pace of bone formation is unparalleled at any other time in adult life; losses owing to inactivity, estrogen deprivation, systemic illness, corticosteroid treatment, and weightlessness or bed rest are slowly and incompletely restored.(1,19–24) The completeness of skeletal recovery has been confirmed by epidemiologic studies of young and postmenopausal women that found no adverse effect of lactation on bone mass, bone density, or hip fracture risk; in several studies, lactation conferred a higher bone mass and lower risk of osteoporosis.(1,25,26)

What is especially puzzling and most clinically relevant is that the factors that stimulate bone formation after weaning are unknown. Repletion of ovarian hormones is likely a factor but not the sole explanation, especially because reproductive-age women who have undergone 6 months of estrogen deprivation therapy lose a trivial amount of BMC but still have a deficit in BMC a year after return of normal ovarian function.(1) We have studied the role of the classic calciotropic hormones and found that mice lacking parathyroid hormone (PTH), calcitonin, or the vitamin D receptor (VDR) are able to stimulate bone formation after weaning and fully restore BMC that was lost during lactation.(6,7,12)

Sustained high levels of PTH (eg, primary or secondary hyperparathyroidism) and PTHrP (eg, hypercalcemia of malignancy, mammary hyperplasia, and normal lactation) are well known to stimulate bone resorption.(27,28) Conversely, short pulses of PTH or PTHrP (such as through the use of once-daily subcutaneous injections) have been shown to stimulate bone formation in humans and rodents, both PTH(1–34) (teriparatide) and PTH(1–84) are clinically approved anabolic treatments for osteoporosis, and PTHrP(1–36) is currently being studied for the same purpose.(29,30) PTH and PTHrP stimulate bone formation by acting on the PTH/PTHrP receptor expressed by osteoblasts.(27,28) Sustained high levels of PTH or PTHrP upregulate osteoblast expression of the receptor activator of NF-κB ligand (RANKL) and downregulate their expression of osteoprotegerin (OPG); the result is stimulation of osteoclast differentiation, osteoclast function, and bone resorption.(31)

The ability of PTH and PTHrP to stimulate bone formation makes them obvious candidates to stimulate bone formation during postweaning recovery. And yet our studies of mice lacking PTH have shown no impairment in the ability of such mice to recover from skeletal losses during lactation.(12) Although mammary-derived PTHrP contributes to the skeletal losses during lactation, this tissue source cannot explain postweaning skeletal recovery because the mammary glands rapidly involute after weaning, and plasma PTHrP levels normally are low or undetectable except during lactation in humans and rodents.(1,2)

Another relevant source of PTHrP in the adult skeleton is preosteoblasts and osteoblasts.(32) Recent studies have shown that PTHrP, in particular osteoblast-derived PTHrP, contributes to the maintenance of adult bone mass(32–34); without it, mice lacking osteoblast-derived PTHrP (*obPthrp* nulls) have significantly reduced bone mass and strength.(32)

We hypothesized that PTHrP is upregulated in bone during postweaning to stimulate bone formation and skeletal recovery, and we used *obPthrp* null mice to test this hypothesis.

## Materials and Methods

### Animal husbandry

A previous report has described in detail how the *Pthrp* gene was deleted using *Cre-lox* technology and the collagen I promoter to drive the *Cre* recombinase.(32) These *obPthrp* mice were backcrossed into the parent strain, C57BL/6, for more than 10 generations and maintained by breeding heterozygous-deleted mice together. Mice were genotyped from DNA extracted from tail clips of newly weaned pups. A Southern blot detected the floxed and normal *Pthrp* allele, whereas polymerase chain reaction (PCR) was used to detect the presence of the *Cre* recombinase; the probes, primers, and conditions have been reported previously.(32,35)

Reproductive studies compared *Pthrp*^flox/flox;^*Cre*^ColI^ (*obPthrp* null) mice with *Pthrp*^+/+^;*Cre*^ColI^ (control or wild-type) mice. To obtain these, *Pthrp*^flox/+;^*Cre*^ColI^ (heterozygous) mice were mated together, and offspring not expressing *Cre* were discarded. The heterozygotes contributed to maintaining the colony, whereas the wild-type (WT) and *obPthrp* null mice were used in experiments.

Experimental mice were mated overnight. The presence of a vaginal mucus plug on the morning after mating marked gestational day 0.5; normal gestation is 19 days. The mice had *ad libitum* access to water and a standard rodent diet containing 1% calcium. All studies were performed with the prior approval of the respective investigator's animal ethics committee (Institutional Animal Care Committee of Memorial University of Newfoundland and the Institutional Animal Care and Use Committee at Yale University).

### Reproductive cycles and data-collection time points

In the studies to determine PTHrP expression in bone during recovery from lactation, weaning was forced at day 12 of lactation by removing the pups from the mother. The remaining studies were completed according to approximate 75-day reproductive cycles. A minimum of two baseline BMC scans and blood/urine collections were completed over 5 to 10 days prior to first mating (prepregnancy interval). Additional BMC scans and sample collections also were done on day 18.5 of pregnancy, day 21 of lactation (39.5 days after the start of pregnancy), and weekly during the postweaning recovery of the skeleton (days 7, 14, and 21 after weaning). Consequently, the mice had an expected mean age of 23 weeks by the end of postweaning recovery.

### Bone mineral content

We measured BMC with the PIXImus 2 Bone Densitometer (GE Lunar, Madison, WI, USA), calibrated daily to a standard phantom (fat 11.9% and BMC 0.063 g). Data were analyzed with PIXImus software (Version 2.1). Anesthesia was induced with isoflurane (Baxter, Mississauga, ON, Canada) and maintained for 5 to 15 minutes with a single intraperitoneal injection of a 5:1 combination of ketamine hydrochloride (Wyeth Animal Health, Guelph, ON, Canada) and xylazine (Bayer, Toronto, ON, Canada). The anesthetized mice were immobilized prone on holding trays with the spine straightened; to maintain reproducibility with less than 0.5% precision error, the head was excluded in all scans. In prior quality-control studies, mice on day 18.5 of pregnancy were scanned immediately before and after the pups were removed by C-section; this determined that the fetal skeletons contributed less than 1% to the apparent maternal BMC and therefore were negligible.(6,36) Whole-body and regional (ie, spine and hind limb) BMC measurements were obtained for each mouse, and the absolute values were normalized to the respective nonpregnant baseline measurements.

### Chemical and hormone assays

Urine and sera were collected in the morning by having mice void into a clean, empty cage; after this, blood was taken from tail veins. Serum or urine calcium, phosphorus, and creatinine were measured with colorimetric assays (Diagnostic Chemicals Limited, Charlottetown, PEI, Canada). PTH was measured with a rodent PTH(1–34) ELISA kit that has a detection limit of 1.6 pg/mL (Immutopics, San Clemente, CA, USA). Calcitriol was analyzed with an EIA kit with a detection limit of 6 pmol/L (Immunodiagnostic Systems, Ltd., Boldon, Tyne and Wear, UK). Bone markers included osteocalcin, assessed by a two-site immunoradiometric assay (Immutopics); propeptide of type 1 collagen (P1NP) by EIA (Immunodiagnostic Systems); and deoxypyridinoline, assessed by the METRA DPD enzyme immunoassay kit (Quidel Corporation, San Diego, CA, USA). Urinary deoxypyridinoline, calcium, and phosphorus were expressed relative to creatinine to correct for variations in urine concentration.

### Biomechanical testing

Tibias were harvested on day 7 after weaning (during rapid bone recovery), stripped of soft tissues, and stored at −20°C. They were thawed to room temperature in PBS for at least 2 hours prior to analysis. Cortical bone strength was assayed using a single-column Instron Series 3340 electromechanical test instrument (Instron, Norwood, MA, USA). In brief, each tibia was immobilized at each end in a fixture, and a load cell with maximum capacity of 10 N of force was positioned 1 cm above the tibial midshaft. The load cell's cross-head descended at 10 mm/minute. The force required to break the tibia (failure) and other biomechanical parameters were detected and recorded automatically by computer.

### RNA extraction

Baseline expression of PTHrP in WT bone was determined in tibias harvested on day 12 of lactation (day of forced weaning) and days 1, 3 and 7 of postweaning recovery. Additional tibias from *obPthrp* null mice and WT siblings were obtained on day 7 of postweaning recovery following a normal 21-day lactation. The growth plates were removed to eliminate cartilaginous tissue. The tibias were snap frozen in liquid nitrogen. Total RNA was extracted and purified using the RNeasy Midi Kit (Qiagen, Valencia, CA, USA). RNA quality was confirmed with the Agilent 2100 BioAnalyzer (Agilent Technologies, Santa Clara, CA, USA).

### Real-time quantitative RT-PCR

We used TaqMan gene expression assays to determine expression of PTHrP in bones with predesigned primers and probes for optimal amplification. Details of conditions and cycle times have been reported previously.(6,37) We used the TaqMan RNA-to-C_T_ 1-Step Kit (Applied Biosystems, Inc., Foster City, CA) in addition to the TaqMan gene expression assays in order to eliminate the need to carry out a separate cDNA synthesis step prior to real-time quantitative RT-PCR. With use of the TaqMan RNA-to-C_T_ 1-Step Kit, the thermal cycler protocol then consisted of a 15-minute cycle at 48°C, a 10-minute cycle at 95°C, followed by 40 cycles of 15 seconds at 95°C and 1 minute at 60°C. We performed all real-time quantitative RT-PCR using the ABI PRISM 7000 Sequence Detection System (Applied Biosystems), as described previously.(37,38) All samples were analyzed in triplicate and in three separate experiments. Relative expression ratios were representative of the threshold cycle (the PCR cycle at which an increase in reporter fluorescence is above a baseline signal) normalized to *glyceraldehyde 3-phosphate dehydrogenase* (*GAPDH*) mRNA.

### Histomorphometry

Tibias were obtained on day 21 of postweaning recovery, stripped of muscle and other soft tissues, fixed in 10% buffered formalin, and embedded in methacrylate. Histomorphometric analysis of 5-µm toluidine blue-stained sections was carried out across the width of the secondary spongiosa beginning 370 µm below the growth plate and extending for 1.11 mm, as described previously, using the Osteomeasure system (Osteometrics, Decatur, GA, USA).(7,39,40)

### Statistical analysis

Data were analyzed using SYSTAT 5.2.1 for Macintosh (SYSTAT, Inc, Evanston, IL, USA). ANOVA was used for the initial analysis; Tukey's test determined which pairs of means differed significantly from each other. Real-time PCR results were analyzed by the 2^–ΔΔ*CT*^ method, where the target and reference are amplified in separate wells.(41) Two-tailed probabilities are reported, and all data are presented as mean ± SE.

## Results

### PTHrP is upregulated in bone during weaning

**Fig. 1:**
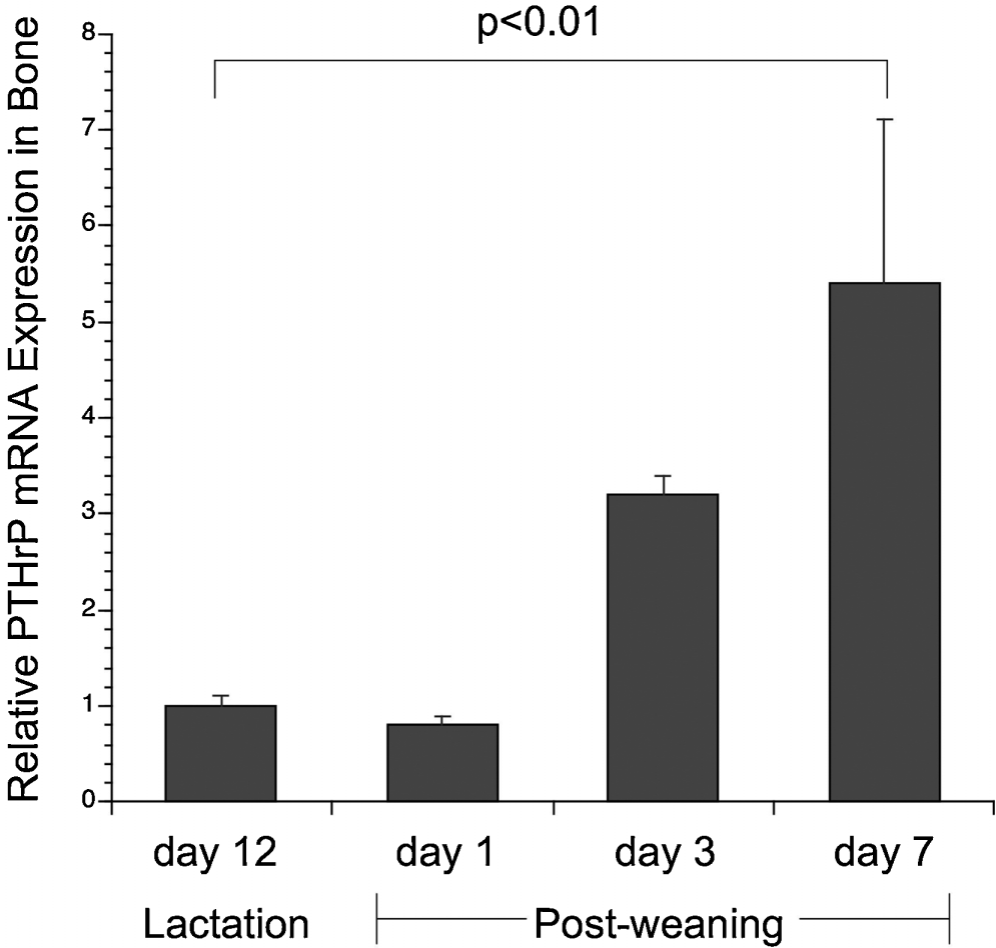
*Pthrp* mRNA expression is upregulated in bone during postweaning recovery. Weaning was forced on day 12 of lactation, and skeletal mRNA was analyzed by real-time quantitative RT-PCR. PTHrP expression increased more than fivefold in bone through the first 7 days after weaning. Values were normalized to *GAPDH* and then expressed relative to the value on day 12 of lactation.

We forced weaning in WT mice on day 12 of lactation and obtained their tibias during postweaning in order to assay *Pthrp* mRNA expression by real-time quantitative RT-PCR. *Pthrp* mRNA expression was similar on day 12 of lactation and the first day after weaning but increased over fivefold by day 7 (Fig. 1). PTHrP is expressed primarily by osteoblasts within bone,(32) and so the increased signal likely was coming from these cells. These results indicate that PTHrP is upregulated in bone during postweaning recovery.

### Bone mineral content excursion in *obPthrp* null mice

The upregulation of PTHrP in bone after weaning indicated that it may stimulate bone formation during this time frame. In addition, prior studies in *Pthrp*^+/–^ mice and in *obPthrp* null mice indicated that osteoblast-derived PTHrP plays a physiologically important role in regulating bone formation and adult bone mass.(32–34) Without osteoblast-derived PTHrP, baseline bone mass and strength are reduced in adult mice.(32) We therefore studied *obPthrp* null mice during full reproductive cycles to determine if lack of osteoblast-derived PTHrP impaired the ability to stimulate bone formation and recover BMC after weaning. Prior published work had shown that PTHrP expression is absent in osteoblasts and preosteoblasts of *obPthrp* nulls.(32) We extracted RNA from tibias (with marrow intact) of *obPthrp* null mice on day 7 of postweaning recovery and found that PTHrP expression was undetectable through 35 cycles of quantitative real-time RT-PCR; relative expression of *Pthrp* mRNA was 0.12 ± 0.01 in *obPthrp* null versus WT mice (*p* < .0001). This indicates not only that PTHrP was eliminated from bone but also that normal expression in marrow is negligible at best.

**Fig. 2:**
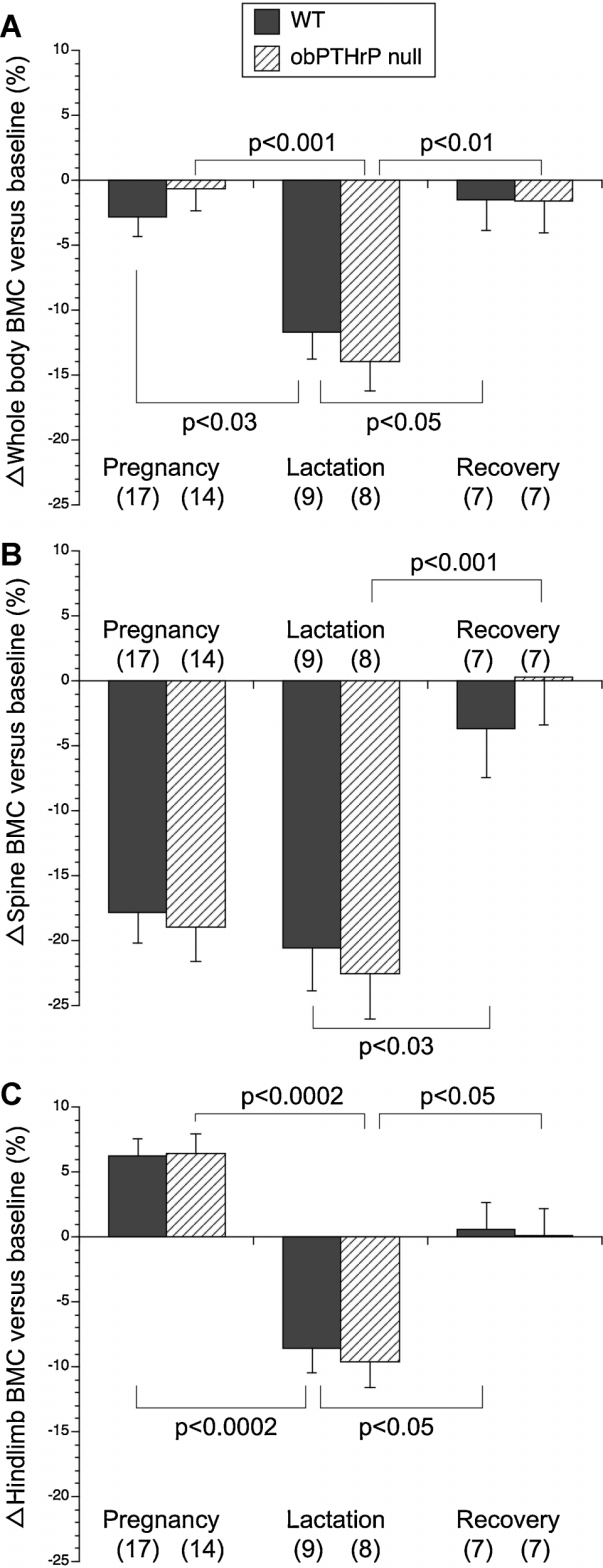
*obPthrp* null and WT mice experience identical changes in BMC during pregnancy and lactation and after weaning. Relative changes in whole-body (*A*), spine (*B*), and hind limb (*C*) BMC versus baseline are depicted on day 18.5 of pregnancy, day 21 of lactation, and day 21 of postweaning recovery. The number of observations is indicated in parentheses.

We next determined changes in BMC in *obPthrp* null mice and WT siblings throughout complete reproductive cycles. At baseline, the whole-body BMC was numerically but not significantly lower in *obPthrp* null mice than in their WT siblings (0.487 ± 0.008 g versus 0.500 ± 0.010 g, *p* = NS). There also was no difference in baseline spine or hind limb BMC (data not shown). These results are similar to the prior published report of *obPthrp* null mice in which the areal bone mineral density (aBMD) was slightly reduced but a more marked reduction in bone mass was evident by micro–computed tomography (µCT) or histomorphometry.(32) By the end of lactation, WT siblings and *obPthrp* null mice lost the same amount of whole-body BMC, and each recovered fully after weaning to a value not significantly different from their respective baselines (Fig. 2*A*). The speed of recovery to baseline was no different between control and *obPthrp* null mice: Recovery was to −7.8% ± 2.1% and −4.2% ± 2.2% of baseline in WT mice at 1 and 2 weeks after weaning and −4.8% ± 2.4% and −2.1% ± 2.4% of baseline at the respective time points in *obPthrp* null mice. The values plotted in Fig. 2*A* were obtained at 3 weeks after weaning.

Skeletal losses are more profound from trabecular bone than from cortical bone during lactation; whole-body BMC largely reflects cortical bone, whereas the spine reflects largely trabecular bone. WT and *obPthrp* null mice lost the same amount of BMC from the spine by the end of lactation, and each recovered fully after weaning with no differences between the two (Fig. 2*B*). Notably, both WT and *obPthrp* null mice lost BMC from the spine during pregnancy. This contrasts with our previous reports of significant gains in BMC during pregnancy in an outbred strain of mice, Black Swiss (Taconic, Germantown, NY, USA).(6,7,36) At the hind limbs of WT and *obPthrp* null mice, there were significant increases in BMC during pregnancy, in keeping with the increased weight bearing. Losses at the hind limb during lactation typically are less than at the spine or whole body, and again there was no difference between WT and *obPthrp* null mice, with full recovery observed in each after weaning (Fig. 2*C*).

Static histomorphometry of the tibia confirmed that the *obPthrp* null mice had lower trabecular bone volume and trabecular number at baseline than WT mice (Table 1). Interestingly, while trabecular bone volume in WT mice was lower 21 days after weaning than baseline bone volume, in the *obPthrp* null mice, bone volume was no different from baseline after 21 days of recovery. There also were no significant differences in osteoblast, osteoid, and osteoclast parameters between genotypes or time points, although the low volume of trabecular bone present in the mice may have limited the ability to detect any differences (Table 1). The lower trabecular bone volume in tibias of postweaning WT mice contrasts with the dual-energy X-ray absorptiometry (DXA) result and suggests that full recovery of tibial microarchitecture did not occur. This is consistent with a recent report that found regional differences in the completeness of recovery of skeletal microarchitecture after weaning, as determined by µCT: Spine recovered fully, whereas femur and tibia had incomplete recovery, but at all three sites, biomechanical strength returned to baseline.(42)

**Table 1:** Static Histomorphometry of the Tibia at Baseline (12 Weeks of Age) and End of Recovery (Day 21 After Weaning or 23 Weeks of Age)

	Trabecular structure			Osteoblast parameters			Osteoclast parameters		
			
Genotype (*n*)	BV/TV %	Tb.Th µm	Tb.N/mm	OS/BS %	Ob.S/BS %	O.Th µm	N.Ob/BPm/mm	OcS/BS %	N.Oc/BPm/mm
WT baseline (3)	3.9 ± 0.5^a^,^b^	22.1 ± 2.8	1.7 ± 0.2^a^,^b^	13.3 ± 1.6	14.9 ± 2.0	0.41 ± 0.05	12.1 ± 0.6	4.9 ± 1.5	2.7 ± 0.6
WT recovery (6)	0.7 ± 0.3^b^	12.1 ± 4.5	0.3 ± 0.1^b^	26.0 ± 4.5	27.7 ± 4.3	0.70 ± 0.17	20.0 ± 3.9	4.4 ± 2.0	2.3 ± 1.0
*obPthrp* null baseline (3)	0.9 ± 0.4^a^	17.8 ± 0.3	0.8 ± 0.1^a^	17.3 ± 8.6	18.8 ± 8.9	0.35 ± 0.05	11.9 ± 6.5	4.8 ± 0.4	2.9 ± 0.4
*obPthrp* null recovery (5)	1.4 ± 0.2	16.1 ± 4.1	0.4 ± 0.2	11.4 ± 1.2	12.6 ± 1.9	0.32 ± 0.08	8.0 ± 1.1	2.8 ± 1.5	1.9 ± 1.0

Abbreviations: BV/TV = trabecular bone volume/total volume; Tb.Th = trabecular thickness; Tb.N = trabecular number; OS/BS = osteoid surface/bone surface; Ob.S/BS = osteoblast surface/bone surface, O.Th = osteoid thickness; N.Ob/BPm = number of osteoblasts per unit bone perimeter; OcS/BS = osteoclast surface/bone surface; N.Oc/BPm = number of osteoclasts per unit bone perimeter.

a *p* < .05 WT versus *obPthrp* null at same time point.

b *p* < .01 WT at baseline versus recovery.

**Fig. 3:**
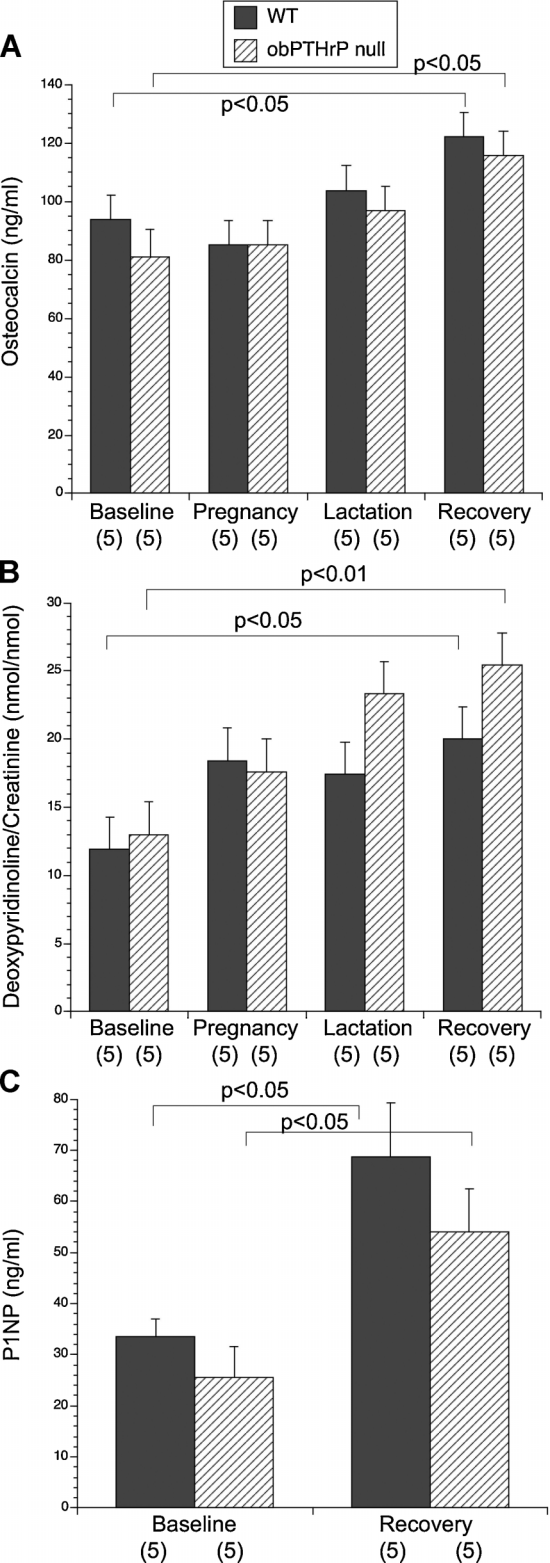
Bone turnover is increased after weaning. The bone markers osteocalcin (*A*), deoxypyridinoline (*B*), and P1NP (*C*) increased equally in WT and *obPthrp* null mice during postweaning recovery compared with the prepregnancy baseline. Depicted here are baseline, day 18.5 of pregnancy, day 21 of lactation, and day 7 of postweaning recovery. The most marked increase in bone resorption occurs during the first 10 days of lactation, but the time point assayed here was day 21 of lactation, when the pups were weaned. The number of observations is indicated in parentheses.

Consistent with the lack of difference in BMC between groups during lactation and after weaning, there also was no difference in bone turnover markers. Osteocalcin, P1NP, and deoxypyridinoline rose significantly during postweaning compared with baseline, but there was no difference between groups (Fig. 3). Importantly, the rise in osteocalcin and P1NP during postweaning is consistent with bone formation that occurs during this interval. Of note is that deoxypyridinoline normally peaks in the first 7 to 10 days of lactation during the interval of greatest bone resorption, but this time point was not measured because the focus in this study was on recovery from lactation. The time point shown in Fig. 3 is the end of lactation, by which point in time bone resorption has lessened greatly.

### Mineral metabolism during lactation and recovery

**Fig. 4:**
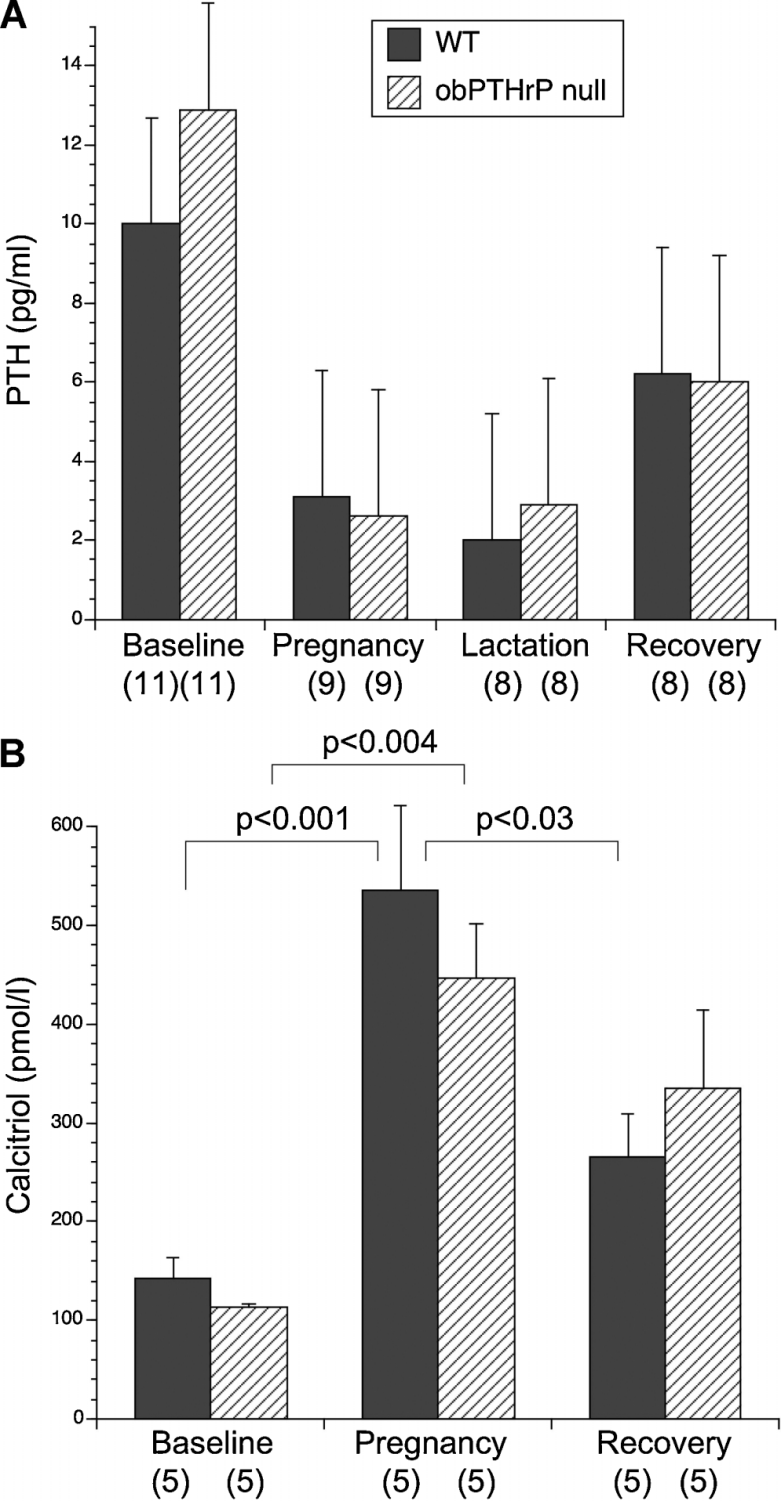
Flux in PTH and calcitriol during reproductive cycles. (*A*) PTH normally becomes suppressed during pregnancy and lactation but rises to baseline during postweaning recovery. This pattern was noted in WT and *obPthrp* null mice, although the changes were not statistically significant. (*B*) Calcitriol tripled during pregnancy, as expected, and then declined during recovery but remained double the baseline value. No significant differences were seen between WT and *obPthrp* null mice. Time points depicted are baseline, day 18.5 of pregnancy, day 21 of lactation, and day 7 of postweaning recovery. The number of observations is indicated in parentheses.

**Fig. 5:**
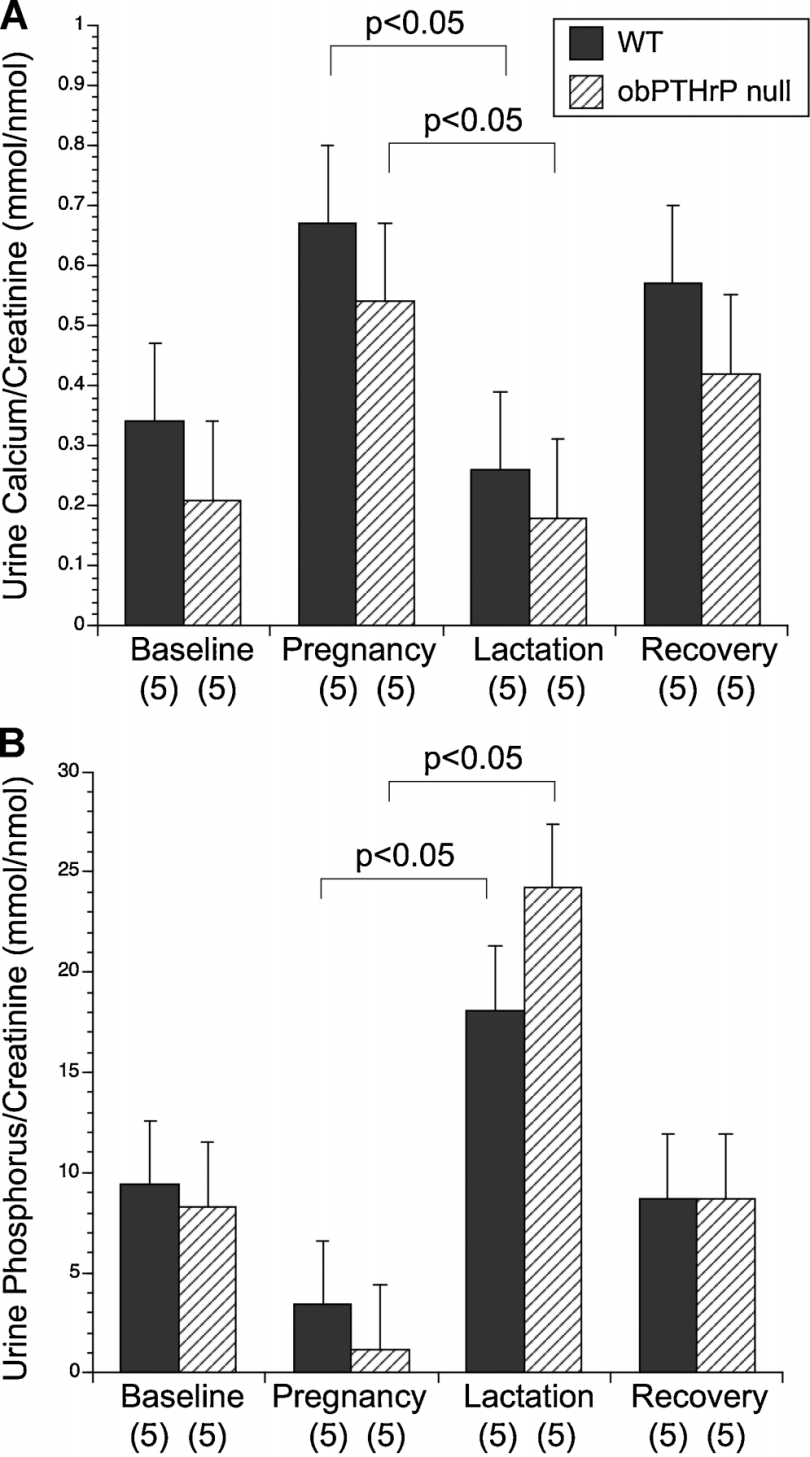
Flux in urinary calcium and phosphorus during reproductive cycles. (*A*) Urinary calcium normally increases during pregnancy and decreases during lactation; the fall during lactation was statistically significant. (*B*) Urinary phosphorus decreased nonsignificantly during pregnancy but increased significantly during lactation as a consequence of enhanced bone resorption and possibly PTHrP-stimulated phosphaturia. In all panels, there were no significant differences between WT and *obPthrp* null mice. Time points depicted are baseline, day 18.5 of pregnancy, day 21 of lactation, and day 7 of postweaning recovery. The number of observations is indicated in parentheses.

We also studied aspects of systemic mineral metabolism during lactation and after weaning to determine if absence of osteoblast-derived PTHrP impaired maternal mineral metabolism or resulted in any compensatory responses. Serum calcium and phosphorus remained unchanged (Table 2). Serum PTH normally falls during lactation and remains low during postweaning recovery, and this is what we observed in *obPthrp* null and WT mice (Fig. 4*A*). Calcitriol showed the expected marked increase during pregnancy and declined significantly during recovery but still remained above baseline with no differences between genotypes (Fig. 4*B*). Urine calcium typically rises during pregnancy owing to increased intestinal calcium absorption (absorptive hypercalciuria) and falls during lactation to prepregnancy values or below. We observed the expected significant decline in urine calcium during lactation, and at all time points WT and *obPthrp* null mice showed similar values with no significant differences between them (Fig. 5*A*). Urine phosphorus increases significantly during lactation as a consequence of accelerated bone resorption and possibly PTHrP-stimulated phosphaturia, and this was found in *obPthrp* null and WT mice with no differences between them (Fig. 5*B*).

**Table 2:** Serum Chemistries at End of Pregnancy and Lactation and Day 7 After Weaning

Genotype (*n*)	Baseline	Pregnancy	Lactation	Recovery
Serum calcium (mmol/L)				
WT (5)	1.97 ± 0.07	2.06 ± 0.08	2.00 ± 0.06	2.04 ± 0.08
*obPthrp* null (5)	1.87 ± 0.11	1.98 ± 0.05	1.99 ± 0.12	2.00 ± 0.06
Serum phosphorus (mmol/L)				
WT (5)	3.07 ± 0.49	2.62 ± 0.26	2.48 ± 0.10	2.93 ± 0.11
*obPthrp* null (5)	2.98 ± 0.35	1.93 ± 0.23	2.91 ± 0.37	2.89 ± 0.20

*Note:* No statistically significant differences were observed.

### Biomechanical testing

The *obPthrp* null mice have been shown previously to have lower bone mass than WT mice at baseline,(32) and this can be expected to increase skeletal fragility. We harvested tibias at baseline and day 7 of postweaning recovery, and we subjected those tibias to the three-point bend test to failure. The tibias of WT and *obPthrp* null mice were not weakened by lactation but absorbed more energy prior to failure when obtained after weaning compared with baseline (Table 3). Tibias from *obPthrp* null mice appeared modestly weaker than those of WT mice at baseline and during postweaning recovery, although the difference was only significant on day 7 of postweaning recovery (Table 3).

**Table 3:** Biomechanical Parameters in Tibias at Baseline (12 Weeks) and Day 7 After Weaning (21 Weeks)

Genotype (*n*)	Ultimate Load (g)	Displacement (µm)	Stiffness (g/mm)	Energy absorbed (g/mm^2^)
WT baseline (8)	1.80 ± 0.06	0.62 ± 0.09	8.27 ± 0.51	0.56 ± 0.03
WT recovery (7)	1.91 ± 0.07^a^	0.66 ± 0.09	8.30 ± 0.31	2.43 ± 0.08^b^,^c^
*obPthrp* null baseline (5)	1.69 ± 0.09	0.63 ± 0.12	8.26 ± 0.51	0.57 ± 0.03
*obPthrp* null recovery (6)	1.60 ± 0.07^a^	0.79 ± 0.10	9.33 ± 0.34	2.03 ± 0.09^b^,^c^

a *p* < .03 WT versus *obPthrp* null at same time point.

b *p* < .002 WT versus *obPthrp* null at same time point.

c *p* < .001 WT at baseline versus recovery.

## Discussion

Enhanced skeletal resorption during lactation is a physiologically important adaptation that enables the mother to provide the necessary amount of calcium to milk. Moreover, this temporary loss of bone mass can have adverse consequences in the short term. A 5% to 10% loss of BMC over 2 to 6 months in humans can reduce the BMC into the osteopenic range or lower depending on the baseline BMC. Some lactating women present with vertebral crush fractures and a very low BMC that was apparently caused by skeletal losses during lactation.(2) In rodents, the BMC losses are more rapid and of greater magnitude than in humans, with the most dramatic being a 55% loss of spine BMC observed in mice that lack the gene encoding calcitonin and calcitonin gene–related peptide α.(6) In humans and all other mammals that have been studied, the skeleton recovers bone mass during postweaning, even in those who fractured(43,44); consequently, lactation is not associated with any long-term risk of skeletal fragility or reduced bone mass.(1,2)

While the mechanism by which skeletal resorption occurs during lactation is reasonably but not fully elucidated, the mechanisms that stimulate bone formation after weaning remain unknown. The postweaning interval is a time of net bone formation that is unequaled at any other time in the adult human, but it bears similarity to comparable loss and recovery of bone mass that occurs in egg-layers and animals that form mineralized antlers and horns.(1,3,45) Evidence described earlier indicates that several classic calciotropic hormones (ie, PTH, calcitriol/VDR, and calcitonin) are not required for the skeleton to restore bone mass after weaning and that recovery of estradiol levels to normal cannot explain the speed or completeness of recovery either. In this study we add to this list by showing that PTHrP is also not required.

Osteoblast-derived PTHrP is necessary for maintenance of normal bone mass because *obPthrp* null mice have low bone mass compared with their WT siblings.(32) This prompted us to consider whether osteoblast-derived PTHrP might stimulate skeletal recovery after weaning. We found that *Pthrp* mRNA was upregulated in bone during the postweaning interval, so we used *obPthrp* null mice to determine if lack of osteoblast-derived PTHrP impaired skeletal recovery after weaning. To our surprise, we discovered that *obPthrp* null mice lactated normally, lost the same amount of BMC as WT mice, and returned to baseline BMC after weaning with no impairment in the magnitude or speed of skeletal recovery. We also found no impact of the absence of osteoblast-derived PTHrP on maternal mineral homeostasis during prepregnancy, lactation, and postweaning. PTH remained suppressed from prepregnancy baseline during pregnancy, lactation, and postweaning recovery, indicating that PTH did not need to upregulate in response to the absence of PTHrP. Calcitriol increased normally during pregnancy and postweaning with no difference between genotypes. We did find that *obPthrp* null mice had reduced trabecular bone volume and strength in the tibias compared with WT siblings, thus reaffirming that PTHrP is needed to maintain adult bone mass and strength.

An important strength of our analytic approach is that sister pairs of WT and *obPthrp* null mice were followed longitudinally by densitometry through 75-day (or longer) reproductive cycles. Consequently, the seven pairs of mice studied at the end of recovery are represented at every time point of Fig. 2 and clearly demonstrate loss of BMC from the whole body, spine, and hind limb, with full recovery after weaning. So too the biochemical analyses represent serial or longitudinal comparisons from mice that contributed to all time points. In contrast, histomorphometry and biomechanical testing are cross-sectional analyses because mice must be euthanized at each time point. The biomechanical testing showed no reduction in tibial strength of either genotype after weaning, whereas the histomorphometric analysis found incomplete recovery of tibial microarchitecture in the WT mice. This is consistent with a recent µCT analysis of normal mice that demonstrated incomplete recovery of tibia and femur but full recovery of spine microarchitecture after weaning and full recovery of biomechanical strength at all three sites.(42)

Our results indicate that osteoblast-derived PTHrP is not responsible for the increase in bone mass that occurs after lactation. It is conceivable that PTHrP still plays a role but that we did not detect a compensatory mechanism that was invoked in its absence. However, since the skeletal losses by the end of lactation in mice are quite large (∼20% of spine BMC in this study) and both genotypes restored this fully within the same postweaning time frame, it seems more likely that PTHrP does not play a significant role in this recovery. Instead, other factors that remain to be elucidated must stimulate bone formation after weaning.

Although we demonstrated that PTHrP expression is upregulated in bone during postweaning recovery and that this increase is obliterated when PTHrP is eliminated from osteoblasts, it remains unclear what role (if any) PTHrP might have in osteoblasts during this time period. An increase in osteoblast number alone does not explain this increase in total PTHrP expression within bone. In separate analyses, we have found that osteoblast numbers at most double in WT mice by day 7 of postweaning recovery (and osteoclast numbers decrease more than 60%),(46,47) whereas in this study we found that PTHrP is upregulated fivefold in WT bone. Also, histomorphometric assessment of rats found a similar doubling of osteoblast numbers over the first 7 days after weaning, accompanied by an 80% decrease in osteoclast numbers.(16,48)

It is notable that during pregnancy, WT and *obPthrp* null mice had no significant change in whole-body BMC but lost a significant amount from the spine and then lost even more during lactation. This pattern differs from what we have reported previously in the outbred Black Swiss strain, in which a 10% to 15% increase in BMC occurs at the whole body and little or no change in BMC at the spine. The WT and *obPthrp* null mice were in the C57BL/6 inbred strain, and this may explain why the excursion in BMC differed across these reproductive time points. This finding underscores that multiple genetic factors are likely involved in regulating bone mass and resorption in response to the demands of pregnancy and lactation. Black Swiss mice gain significant BMC in advance of the demands of lactation, whereas C57BL/6 mice lose BMC during pregnancy; both experience a similar net loss of about 20% of BMC by the end of lactation. Black Swiss mice also maintain a higher ionized calcium and BMC than C57BL/6 mice,(6,7,49) so these two strains of mice may differ with respect to one or more unknown genes that are pertinent to calcium and bone homeostasis.

Overall, the results of this study of *obPthrp* null mice have yielded the important finding that PTHrP is not required for skeletal recovery after lactation. Taken together with our previous studies in PTH, VDR, and calcitonin/calcitonin gene–related peptide α knockout mice, these results emphasize that factors other than the known calciotropic hormones are controlling the recovery of bone mass after lactation. Understanding how this interval of rapid and substantial bone formation is regulated may lead to the discovery of novel factors that stimulate bone formation and that might be exploited to treat disorders of low bone mass and skeletal fragility pharmacologically. Moreover, the phenotypic differences between Black Swiss and C57BL/6 strains of mice may hint at important genetic differences that are important for the regulation of calcium and bone metabolism.

In conclusion, PTHrP is upregulated in bone during postweaning skeletal recovery, and it is required to maintain adult bone mass and strength. However, osteoblast-derived PTHrP is not required for the skeleton to recover from 20% losses of BMC induced by lactation. The longitudinal DXA measurements definitively show loss of bone mass at cortical and trabecular sites, with full recovery achieved after weaning at all sites. The biochemical (ie, calcium, PTH, calcitriol, and bone markers), biomechanical (ie, three-point bending test), and mRNA expression data (fivefold upregulation of PTHrP in WT bone that is eliminated in the *obPthrp* null) make very clear that loss of osteoblast-derived PTHrP does not compromise the ability of the skeleton to recover after weaning and that calcitriol or PTH do not upregulate to compensate for loss of PTHrP. The factors that regulate bone formation after weaning remain to be identified.
